# Transgenerational effects of in ovo stimulation with synbiotic and choline on gonadal tissue across three generations

**DOI:** 10.1038/s41598-025-16387-6

**Published:** 2025-08-22

**Authors:** Mariam Ibrahim, Ewa Grochowska, Marek Bednarczyk, Katarzyna Stadnicka

**Affiliations:** 1 Faculty of Health Sciences, Collegium Medicum, Nicolaus Copernicus University, Bydgoszcz, Poland; 2 PBS Doctoral School, Bydgoszcz University of Science and Technology, Bydgoszcz, Poland; 3 Department of Animal Biotechnology and Genetics, Bydgoszcz University of Science and Technology, Bydgoszcz, Poland

**Keywords:** Choline, DNA methylation, Gene expression, Gonads, in ovo stimulation, Transcriptome, Transgenerational effect, Animal biotechnology, Next-generation sequencing, RNA sequencing

## Abstract

Epigenetic mechanisms play a pivotal role in hereditary processes, shaping phenotypic outcomes across generations. This study investigates the transgenerational impacts of in ovo injection of bioactive substances on gene expression and DNA methylation in the male gonads using the Green-legged Partridgelike chickens as a model organism. Synbiotic PoultryStar^®^ (Biomin; PS) and choline were injected in ovo on the 12th day of egg incubation. In the F1 generation, three groups were established: (1) control (C, 0.9% physiological saline); (2) PS synbiotic (SYN, 2 mg/embryo); and (3) PS synbiotic combined with choline (SYNCH, 2 mg/embryo of synbiotic and 0.25 mg/embryo of choline). In subsequent F2 and F3 generations, groups SYN and SYNCH were further divided into two subgroups each: (A) only injected in F1 embryos (SYNs and SYNCHs); and (B) repeatedly injected in every generation (SYNr and SYNCHr). At 21 weeks post-hatching, gonadal tissues were sampled from F2 and F3 male chickens for transcriptomic and reduced representation bisulfite sequencing (RRBS). Synbiotic alone produced minimal and diminishing changes in gene expression across generations. In contrast, the single co-administration of synbiotic and choline in F1 embryos (SYNCHs) led to 1,897 differentially expressed genes (DEGs) and 786 differentially methylated regions (DMRs) in F3. Repeated administration across generations (SYNCHr) resulted in an even greater number of DEGs (2,804) and DMRs (2,880) in F3, reflecting a cumulative exposure effect. DEGs in SYNCH groups were mainly enriched in pathways related to cytoskeletal organization and extracellular matrix. In SYNCHs, methylation changes were associated with TGF-beta signaling, whereas SYNCHr showed additional enrichment in Wnt signaling, focal adhesion, and adipocytokine signaling pathways. Integrative analysis revealed coordinated changes in gene expression and DNA methylation, particularly in the F3 generation, identifying 37 genes (47 DMRs) in SYNCHs and 194 genes (306 DMRs) in SYNCHr. This study highlights the potential of prenatal epigenetic interventions to induce gene expression and DNA methylation changes across generations in reproductive tissues.

**Supplementary Information:**

The online version contains supplementary material available at 10.1038/s41598-025-16387-6.

## Introduction

The impact of epigenetic information extends beyond mitotic cell-to-cell inheritance to include meiotic intergenerational and transgenerational inheritance^1^. It is widely recognized that genetic information can undergo epigenetic reprogramming in both the maternal and paternal germlines, potentially leading to inherited phenotypic alterations in offspring^2^. Nutrition plays a significant role as one of the primary external influencers of epigenomes^3^. Nutriepigenetics, the study of how dietary factors influence gene expression through epigenetic mechanisms, unveils a new layer of complexity in understanding the interplay between diet, the gut microbiome, and host health^4^. Interventions with bioactive substances, such as synbiotics and choline supplementation, known for their ability to modulate the gut microbiota, may exert nutriepigenetic effects, ultimately impacting the development and long-term health of the host organism^5,6^. Building on this, early nutritional reprogramming through such nutriepigenetic factors offers a promising avenue for identifying specific epigenetic changes linked to growth and metabolic outcomes^7^. This line of research has been made feasible by advancements in in ovo technology, which allows for the precise administration of various substances, including nutrients, hormones, vaccines, prebiotics, probiotics, and synbiotics as early as day 12 of egg incubation^8^. Diverse lines of evidence converge to suggest that epigenetic markers hold the capacity to be transmitted from parents to offspring through gametes^9,10^. In mammals, research has revealed that the dietary habits of progenitors can influence the inherited epigenetic information passed on to the next generation^11^. One of the pioneering studies linking molecular epigenetic alterations to transgenerational disease inheritance in mammals investigated the impact of administering the agricultural fungicide vinclozolin to pregnant rats^12^. Subsequent generations, F1 to F4, exhibited reproductive abnormalities including elevated testicular germ cell apoptosis and reduced sperm motility. These transgenerational phenotypes were found to be associated with alterations in DNA methylation within sperm cells^12^. Leroux et al. were pioneers in demonstrating transgenerational inheritance in birds, showing that the embryonic environment can influence the phenotype of offspring up to three generations later in quail^13^. Sexual maturity of females, adult body weight and behavioral traits were affected by a single injection of genistein at the onset of egg incubation of the first generation^13^. Similarly, in ducks, providing a diet deficient in methionine resulted in grand-offspring with modified weight gain and metabolic parameter alterations^14^.

In the present study, we used the Green-legged Partridgelike chicken, which is a slow-growing breed, primarily distinguished by its low environmental and nutritional demands, to investigate the transgenerational impact of bioactive substances on male germline. Unlike commercial poultry lines, this breed has undergone limited selective breeding^15^. As an outbred population, it serves as a valuable model for transgenerational epigenetic studies, as it may be more sensitive to epigenetic modifications compared to inbred strains^16^. The knowledge about the transgenerational impact of nutriepigenetic factors in the male gonads remains limited despite their importance in inheritance. While both male and female gonads serve as primary reproductive organs for gamete production, the yolk from the female gonad potentially contains additional factors contributing to intergenerational and transgenerational inheritance compared to the male gonad^17^. Consequently, the male gonad presents an ideal target for studying the direct effects of epigenetic stimulation on transgenerational inheritance. Observing changes in gene expression patterns within gonads is particularly intriguing since these organs are known to exhibit lower metabolic activities compared to active organs, e.g., the liver^17^.

Although extensive research in mammalian models has documented evidence of germline inheritance of epigenetic markers in response to nutritional stimuli^18^our understanding of the intergenerational and transgenerational mechanisms underlying prenatally induced epigenetic stimulations and their impacts in chickens, particularly within less-explored tissues such as the gonads, remains incomplete. The present study aims to investigate the transgenerational effects of in ovo injection of bioactive substances (synbiotic and choline) on gene expression and DNA methylation within the male gonads of Green-legged Partridgelike chickens.

## Materials and methods

### Ethical consideration

All experiments were performed in accordance with relevant guidelines and regulations. Approval for the experimental protocols was granted by the Local Ethical Committee for Animal Experiments in Bydgoszcz, Poland, under Approval No. 15/2022 on 20.04.2022, Directive 2010/63/EU and Regulation (EU) 2019/1010. The study is reported in accordance with ARRIVE guidelines^19^ (https://arriveguidelines.org).

### Birds and experimental design

The experiment was conducted across three successive generations of Green-legged Partridgelike chickens, with 300 eggs allocated per generation across treatment and control groups. Fertilized eggs obtained from F0 hens were incubated in standard conditions in a commercial hatchery, Wagrowiec Poland (37.5 °C, 55% relative humidity, turned every two hours, for 18 days, then in the hatcher for 3 days at 36.9 °C, 65% relative humidity). The selection of choline source and dosage, along with the synbiotic dosage, was based on hatchability results reported in our previous manuscript^20^.

**Fig. 1:**
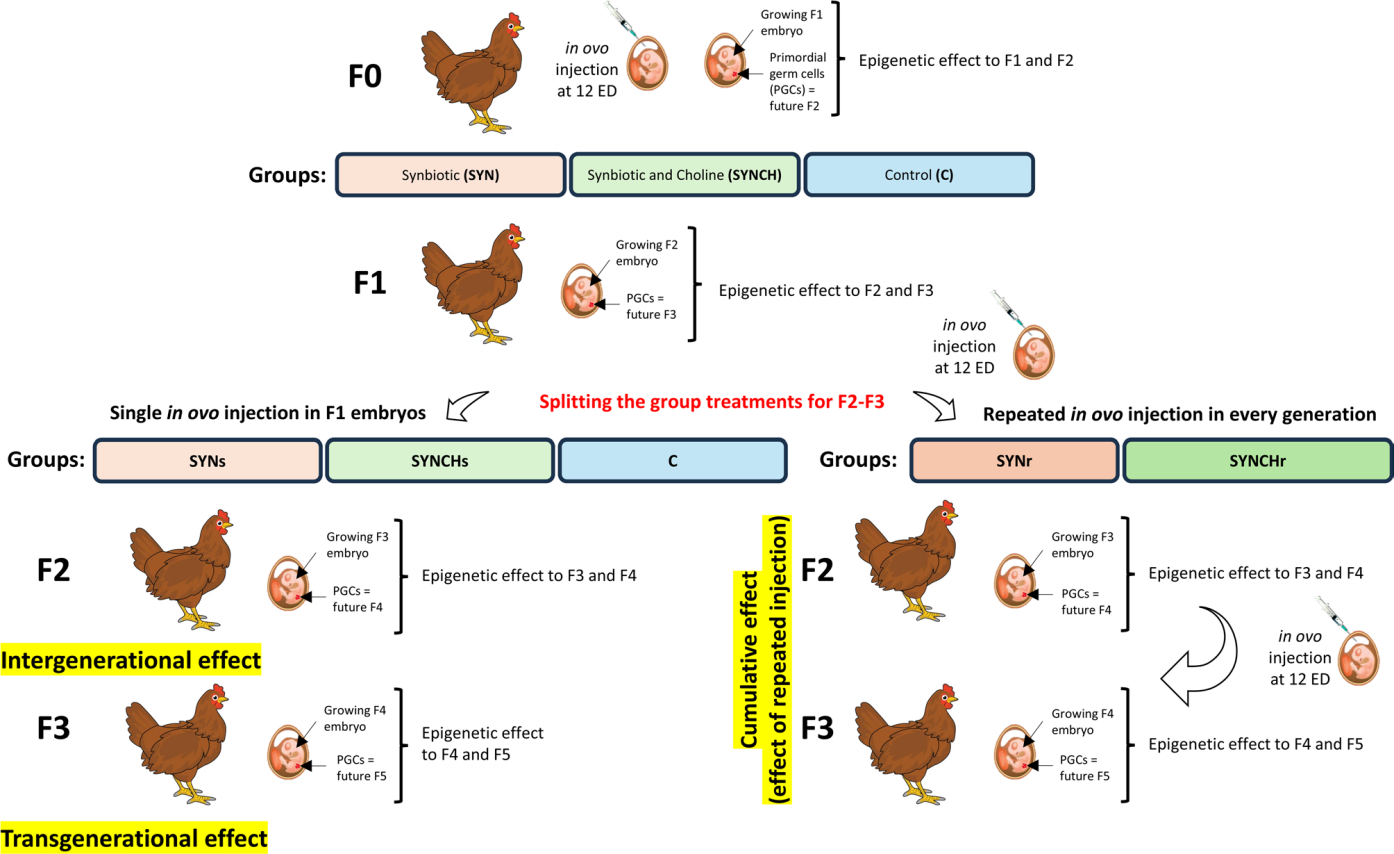
The experimental design of the study. Three experimental groups were established at F1: synbiotic (SYN), synbiotic with choline (SYNCH) and the control group (C, 0.9% physiological saline). The SYN and the SYNCH groups were further split into two groups in F2 and F3, such that two new groups were formed: repeatedly injected synbiotic (SYNr) group and repeatedly injected synbiotic with choline (SYNCHr) group. Additionally, the original SYN and SYNCH groups continued with the only single-injection established in F1, referred to as the SYNs and SYNCHs groups, respectively. SYNs and SYNCHs groups are designed to study the transgenerational effects in F3 chickens. SYNr and SYNCHr groups are designed to study the cumulative effects due to repeated stimulation.

The experimental design is illustrated in Fig. 1 and described in detail in our previous studies^20,21^. In brief, on the twelfth day of embryonic development, after candling, eggs with viable F1 embryos were randomly divided into the following three experimental groups: (1) synbiotic group (SYN) injected with a single dose of synbiotic (PoultryStar^®^ sol^US^, Biomin GmbH, Herzogenburg, Austria; further referred to as PS); 2 mg/embryo suspended in 0.2 mL of physiological saline (NaCl)); (2) synbiotic and choline group (SYNCH) injected with a single dose of the PS synbiotic (2 mg/embryo) and choline (0,25 mg/embryo, Sigma-Aldrich, Saint Louis, MA, USA, cat. no. C7527) suspended in 0.2 mL of NaCl; (3) control group (C) injected with 0.2 mL of NaCl (0.9%). The rearing scheme was continued till F3 generation. In F2 and F3, the treatment groups were split into four, such that two groups were continuously bred without receiving any further injections in F2 and F3. These groups were designated as SYNs (received a single dose of synbiotic in F1 embryos), and as SYNCHs (received a single dose of synbiotic combined with choline in F1 embryos). For the other two groups, the injection of synbiotic and synbiotic with choline was repeated in every generation. These groups were referred to as SYNr and SYNCHr, respectively. The PS synbiotic preparation administered in ovo included a prebiotic (inulin) and a probiotic mixture of 4 microbial strains (5.0 × 10^9^ CFU/g) selected from 4 different sections of the poultry gastrointestinal tract: *Pediococcus acidilactici* isolated from the cecum, *Bifidobacterium animalis* from the ileum, *Enterococcus faecium* from the jejunum and *Lactobacillus reuteri* from the crop. Chickens were reared under semi-intensive rearing conditions in floor pens, *n* = 150 birds divided into five experimental groups (30 birds/group), in two rearing replicates per group per generation. Birds were kept in pens, with a bedding made of chopped wheat straw, enriched with perches. The reared birds were fed a commercial diet free from antibiotics, probiotics and prebiotics, purchased from a feed company (Golpasz, De Heus, Golub-Dobrzyń, Poland) and had a free access to fresh water. The laying hens received feed prepared directly on the farm, based on 75% winter wheat and 25% concentrate for laying hens from De Heus Polska (Manufacturer’s code: 1957 - HD660 × 00 S-W00).

## Sample preparation

Testicular samples were collected from 21-week-old male chickens (*n* = 6). For RNA isolation, samples were fixed in RNAlater buffer (ThermoFisher, Waltham, MA, USA, cat. no. AM7021). These samples were then stored at − 80 °C until later usage. For DNA isolation, separate portions of the same tissue samples were immediately placed on dry ice and then stored at − 20 °C. Metal beads (2.4 mm, cat.no 10032-370, OMNI International USA) were used to homogenize the tissues. RNA was isolated using the GeneMATRIX Universal RNA Purification Kit (EURx, Gdańsk, Poland, cat.no. E3598) following the instructions provided by the manufacturer for animal tissues with the use of RNA Extracol reagent (EURx, Gdańsk, Poland, cat. no. E3700). RNA integrity was evaluated on an Agilent Bioanalyzer 2100 system (Agilent Technologies, Santa Clara, CA, USA) using an RNA Nano 6000 Assay Kit RNA (Agilent Technologies, Santa Clara, CA, USA). In parallel, DNA was isolated using the Tissue DNA Purification Kit (EURx, Gdańsk, Poland, cat. no. E3550), following the manufacturer’s protocol. DNA concentration was measured using a Qubit 4 Fluorometer (Invitrogen™, Thermo Fisher Scientific, Waltham, MA, U.S., Cat. No. Q33238) with the Qubit dsDNA BR Assay Kit (ThermoFisher Scientific, Waltham, MA, USA, Cat. No. Q32850). DNA integrity was evaluated by agarose gel electrophoresis.

## RNA-sequencing and analysis

A total of 30 RNA-seq libraries (*n* = 15 per generation (F2 and F3), *n* = 3 per group) were prepared using the Novogene NGS Stranded RNA Library Prep Set (PT044, Novogene, Cambridge, UK). Sequencing was conducted at a depth of 20 M per sample on the Illumina Novaseq6000 platform by Novogene (Cambridge, United Kingdom), using a 150 paired-end sequencing kit for data generation. Quality control assessment of the raw sequencing data was performed using FastQC v0.12.1 (https://www.bioinformatics.babraham.ac.uk/projects/fastqc/)^22^. Reads underwent a trimming process using the fastp tool v1.0.1^23^. The Q20, Q30, and GC contents of the clean data were analyzed. Following data preprocessing, the paired-end reads were mapped to the chicken genome (Gallus gallus genome assembly GRCg6a (galGal6), Genome Reference Consortium [GCA_000002315.5 GCF_000002315.6]) using STAR 2.7.11b software^24^. Differential expression analysis was conducted using DESeq2 (version 1.48.1)^25^ on RStudio (2025.5.0.496)^26^. The raw counts were normalized using the DESeq2 package. Defined differentially expressed genes (DEGs) were called using adjusted p-value less than or equal to 0.05 and a log2 fold change cutoff of less than or greater than 0.58. Upregulated genes were determined by a log2 fold change greater than 0.58 and an adjusted p-value of ≤ 0.05, while downregulated genes were identified by a log2 fold change less than 0.58 and an adjusted p-value of ≤ 0.05. Enrichment analysis for Gene Ontology (GO) and Kyoto Encyclopedia of Genes and Genomes (KEGG, developed by Kanehisa Laboratories)^27–29^ pathways was performed using clusterProfiler^30^. Multiple testing correction was applied using the Benjamini-Hochberg (BH) method (pAdjustMethod = “BH”). Significance thresholds were set at a p-value ≤ 0.05 and a false discovery rate (q-value) ≤ 0.10. Only terms and pathways with at least three implicated DEGs were considered.

## Validation of sequencing data by quantitative reverse transcription-polymerase chain reaction (RT-qPCR)

To verify the reliability of the RNA sequencing data, five up- and five downregulated DEGs in SYNCH groups in F3 generation were selected for RT-qPCR analysis. Six biological replicates were performed for each sample. The cDNA was prepared using the smART First strand cDNA Synthesis kit (Eurx, Gdańsk, Poland, cat.no. E0804). The cDNA was amplified by real time qPCR with the primers listed in Table 1. Primers were designed using Primer Blast^31^. The reactions were performed in a 20 µL volume containing 50 ng cDNA; 0.25U UNG (uracil-N-glycosylase); and 15 pmol of each forward and reverse amplification primer in 1 × SG qPCR master mix (Eurx, Gdańsk, Poland, E0401). Thermocycling conditions for real time qPCR were as follows: 1 cycle for UNG pre-treatment at 50 °C for 2 min, 1 cycle for initial denaturation at 95 °C for 10 min; and 40 cycles of 94 °C for 15 s, 60 °C for 30 s, and 72 °C for 30 s. Melting-curve profiles were analyzed for all amplicons using the following thermal conditions: 95 °C for 5 s, 70 °C for 5 s, and then a gradual temperature increase to 95 °C at a ramp rate of 0.5 °C/5s. Amplification was performed in CFX Opus 96 real-time PCR system (BIO-RAD, CA, USA). The Pffafl (or standard curve) method was used to analyze the relative expression levels of the studied genes^32^. SRplot was used to visualize the PCR vs. RNA-seq expression double Y axis plot^33^. Pearson’s correlation analysis was performed to evaluate the linear association between log2 fold changes obtained from RNA-seq and qPCR experiments. The correlation coefficient (r), corresponding p-value, and 95% confidence intervals were computed using RStudio (2025.5.0.496)^26^. Statistical significance was assessed at a threshold of *p* ≤ 0.05.

**Table 1:** Primers for RT-qPCR.

	Gene symbol	Gene name	Direction	Primer sequence	Reference
Upregulated genes	SOSTDC1	Sclerostin domain containing 1	Forward	CATTCACTTCTACGGCTTACTCC	This study
			Reverse	CAACTTGAACGCGATTGTTACGG	
	RGS2	Regulator of G-protein signaling 2	Forward	ACCACACCTACTTCAGACCTTC	This study
			Reverse	GTTCTCTTCGCAGAACTCAGAC	
	ELOVL3	ELOVL fatty acid elongase 3	Forward	AAGTCCTGGAACTGGGTGATAC	This study
			Reverse	CACCAGACAACATCTCCTTGTAG	
	STAR	Steroidogenic acute regulatory protein	Forward	AGGAGAAGCCCTTCAGCGAGA	This study
			Reverse	CACTTTGTCTCCGTTGTCCGCC	
	IL21R	Interleukin 21 receptor	Forward	ACATGCAGTGTCTGCGGTCC	This study
			Reverse	GGTTCTGACTGGATGTCCTTGCC	
Downregulated genes	CKMT2	Creatine kinase, mitochondrial 2	Forward	GGTCGATCAGAGGTGGAACT	This study
			Reverse	CAAACTGTGGCAATGGTGGT	
	C1orf158	Chromosome 1 open reading frame 158	Forward	CGAGGAGCCGACATTTGGTA	This study
			Reverse	TAGTTGGTGGGTGGTTCCAG	
	NME4	NME/NM23 nucleoside diphosphate kinase 4	Forward	ATGCACGTCAGCAGGAACG	This study
			Reverse	TCCCTTTGGAACCAGAAGCC	
	SPERT	Spermatid associated	Forward	CAACTCCCACAAGCAGTCCTAATC	This study
			Reverse	GCTTTGTACACCGTGTGCTCTG	
	NEUROD1	Neuronal differentiation 1	Forward	GCTGAGAACGGAGGCGCT	This study
			Reverse	GTCCTCCTCCTTCTTGTCGG	
Reference genes	PPIA	Peptidylprolyl isomerase A	Forward	CCAACCCCGTCGTGTTCTTC	^34^
			Reverse	GTTATGGGCACCTTGTCAGCG	
	ACTB	Actin beta	Forward	TGAACCCCAAAGCCAACAGAG	^35^
			Reverse	TCACCAGAGTCCATCACAATACCA	

### Reduced representation bisulfite sequencing (RRBS) library preparation

RRBS libraries were prepared using the Zymo-Seq RRBS Library Kit (Irvine, California, U.S., cat. no. D5461) following the manufacturer’s protocol. A total of 18 libraries were prepared from the control, SYNCHs and SYNCHr groups in F2 and F3 generations (*n* = 3 per group). A total of 300 ng of genomic DNA was utilized for library preparation with 2% spike-in using *E.coli* genomic DNA (5ng/µl). The concentrations of the prepared libraries were assessed using Qubit 4 fluorometer (Invitrogen™, Thermo Fisher Scientific, Waltham, MA, U.S., Cat. No. Q33238) with Qubit 1X dsDNA HS Assay Kit (Invitrogen™, Thermo Fisher Scientific, Waltham, MA, USA; Cat. No. Q33230). Libraries were validated using the Agilent Bioanalyzer 2100 system (Agilent Technologies, Santa Clara, CA, USA) with the Agilent DNA 1000 Kit (Agilent Technologies, Santa Clara, CA, USA, cat. no. 5067 − 1504).

## RRBS-sequencing and bioinformatic analysis

Sequencing was performed on the AVITI platform (Element Biosciences, San Diego, CA, USA) using a 75-cycle paired-end sequencing kit by Genomed (Warszawa, Poland). FastQC v0.12.1 was used to assess the raw sequencing data’s quality control^22^. Reads were trimmed with Trim Galore (https://github.com/FelixKrueger/TrimGalore). Bismark (Bisulfite Read Mapper and Methylation Caller software, version v0.24.2, https://www.bioinformatics.babraham.ac.uk/projects/bismark/) was used to map the reads to the chicken genome (GRCg6a (galGal6))^36^. Differential methylation analysis was performed using the DSS package^37^ in RStudio (version 2025.5.0.496)^26^. A minimum coverage threshold of 10 reads per CpG site was applied. CpG methylation levels were compared between control and treatment groups using DMLtest() with smoothing enabled to improve robustness. Differentially methylated loci (DMLs) and differentially methylated regions (DMRs) were identified with a minimum methylation difference of 20% and p-value threshold of 0.05. DMRs were annotated using the ChIPseeker package^38^employing the TxDb.Ggallus.UCSC.galGal6.refGene transcript annotation database. The transcription start site (TSS) region was defined as ± 3 kb. Genes associated with DMRs (differentially methylated genes, DMGs) were subjected to GO and KEGG^27–29^ enrichment analysis using the clusterProfiler package^30^ with parameters set to pvalueCutoff = 0.05, qvalueCutoff = 0.10, pAdjustMethod = “BH”. To investigate the relationship between DNA methylation and gene expression, DEGs from RNA-seq analysis were matched with genes associated with DMRs based on shared gene identifiers (ENTREZID). Overlap was determined by comparing DMR-annotated gene IDs with DEGs mapped to ENTREZID using the org.Gg.eg.db annotation package.

## Results

In this study, slow-growing Green-legged Partridgelike chickens were used as a model organism to study the effects of single and repeated in ovo stimulation with potential dietary epigenetic modulators on gonadal tissues across three generations. Groups SYNs and SYNCHs were designed to investigate the transgenerational impact (in F3 generation) of synbiotic alone and with choline following a single in ovo stimulation in F1 embryos. Furthermore, groups SYNr and SYNCHr, underwent in ovo stimulation in every generation, aimed to explore the cumulative effects of repeated stimulation. We presented the resulting changes in gene expression and DNA methylation patterns in the male gonads of F2 and F3 following in ovo stimulation with bioactive compounds acting as potential epigenetic modulators.

### Summary of RNA-seq data

A summary of RNA-seq data quality is provided in Supplementary File S1 (Table S1). The number of raw reads per sample ranged from 19,690,535 to 58,384,722 across all F2 and F3 group samples. After trimming low-quality reads and adapters, the number of clean reads ranged from 19,418,168 to 57,573,407 per sample. Across all samples, over 97% of bases had a quality score of Q20, and over 92% had a quality score of Q30, indicating high sequencing accuracy. GC content varied between 48% and 51%. Clean reads were mapped to the chicken reference genome assembly galGal6 (GRCg6a), and the mapping summary is provided in Supplementary File S1 (Table S1).

### Gene expression changes associated with nutriepigenetic factor supplementation

Using datasets derived from uniquely mapped reads, totaling 25,466 identified genes, differential expression analysis was performed, identifying genes with statistically significant changes in expression (|log2 fold change| threshold = 0.58, adjusted p-value of ≤ 0.05).

**Fig. 2:**
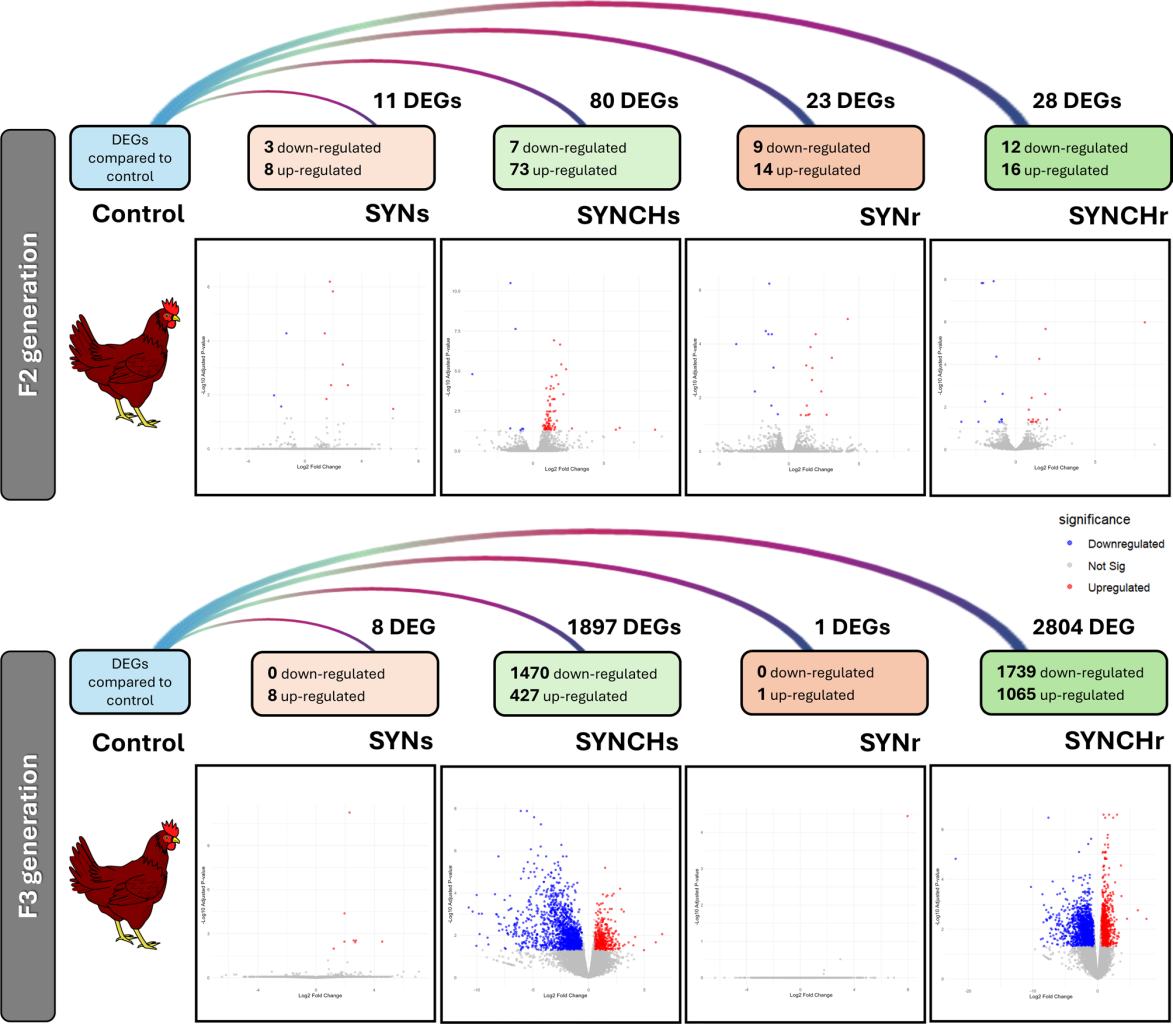
Differentially expressed genes (DEGs) identified in F2 and F3 generations. The diagram summarizes the number of DEGs detected in each experimental group compared to the control. Volcano plots illustrate the expression profiles, where red dots indicate upregulated genes, blue dots indicate downregulated genes, and grey dots represent non-significant genes. DEGs: differentially expressed genes; C: control; SYNs: group of single injection of synbiotic in F1; SYNr: group of repeated injection of synbiotic in F1-F3; SYNCHs: group of single injection of synbiotic with choline in F1; SYNCHr: group of repeated injection of synbiotic with choline in F1-F3.

Figure 2 shows the discrepancy in the number of DEGs when comparing treatment groups with the control in both F2 and F3 generations. The identified DEGs in the gonads across all comparisons are provided in Supplementary file S2. Groups treated with PS synbiotic alone, SYNs and SYNr, showed higher numbers of DEGs in F2 than in F3. There were 11 DEGs for SYNs and 23 DEGs for SYNr in F2 generation, whereas this count was reduced to 8 and 1 DEG for SYNs and SYNr in F3 generation, respectively. Conversely, in the PS synbiotic plus choline-treated groups, we observed an inverse trend. In SYNCHs and SYNCHr in F2, we identified 80 and 28 DEGs, respectively, which markedly escalated to 1,897 and 2,804 DEGs for SYNCHs and SYNCHr in F3, respectively. Notably, administering synbiotic and choline together resulted in a greater number of affected genes compared to PS synbiotic supplementation alone. The results obtained provide compelling evidence that synbiotic supplementation alone, whether administered as a single injection in F1 (SYNs) or repeatedly in F1, F2 and F3 (SYNr), exhibited minimal effect, which even diminished by the F3 generation. However, when synbiotic supplementation was combined with choline, the impact became more pronounced.

In F2, the SYNCHs group shared only 3 overlapping DEGs with SYNCHr, while the SYNs and SYNr shared just one DEG. No common DEGs were found between SYNs and SYNr in F3. In contrast, SYNCHs and SYNCHr in F3 showed substantial overlap, sharing 1,339 DEGs. Across generations (homologous groups in F2 and F3), one common gene was found in SYNs, none in SYNr, 14 in SYNCHs, and 6 in SYNCHr. Overlapping DEG lists are provided in Supplementary File S3.

### Functional clustering by gene ontology (GO)

**Fig. 3:**
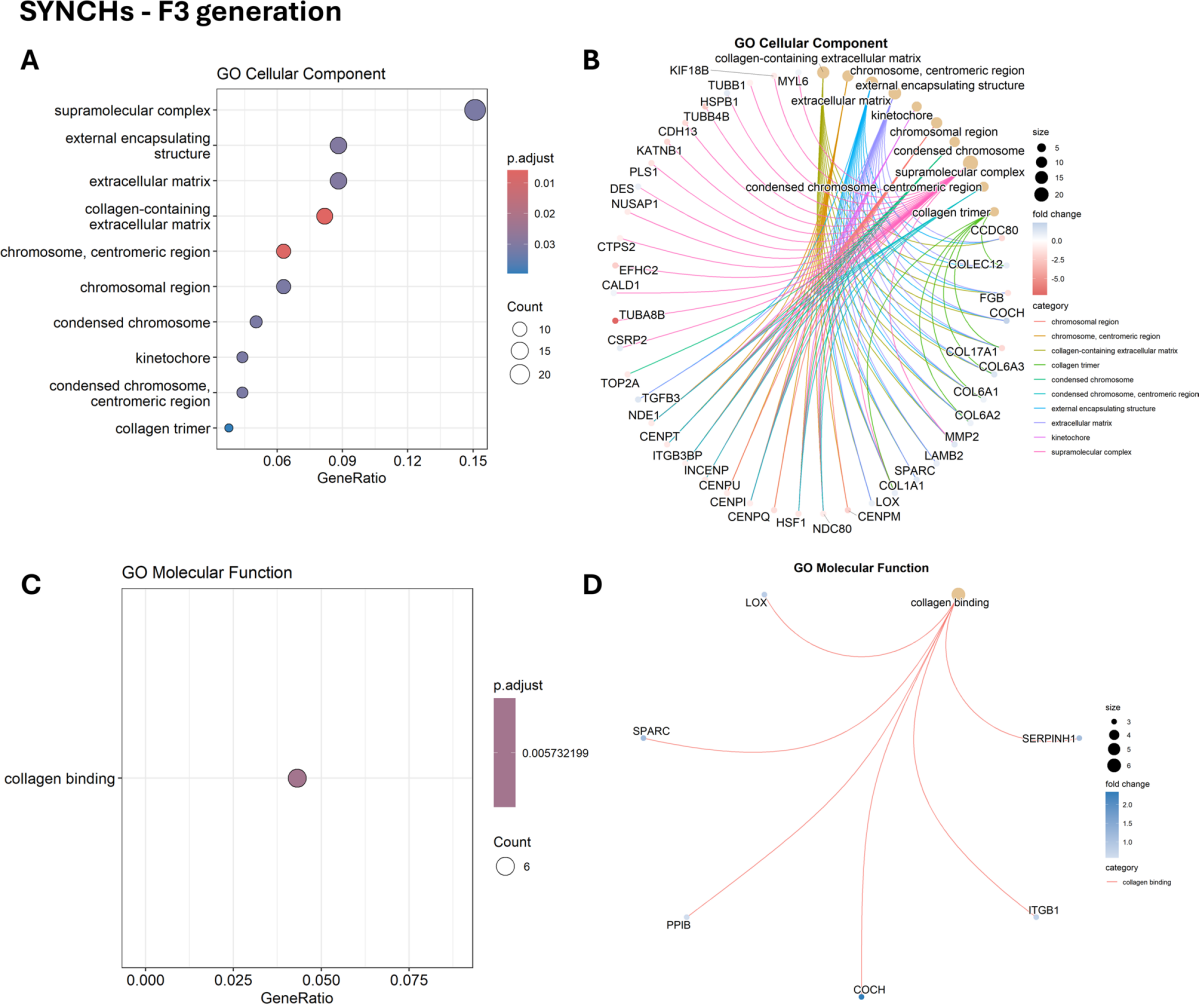
Gene ontology (GO) enrichment analysis of differentially expressed genes (DEGs) in SYNCHs compared to control in F3 generation. (**A**, **C**) Dot plots showing the top 10 enriched GO terms (cellular components and molecular functions, respectively). (**B** and **D**) Cnet plots showing the relationship between the DEGs and GO terms for the enriched cellular components and molecular functions, respectively.

**Fig. 4:**
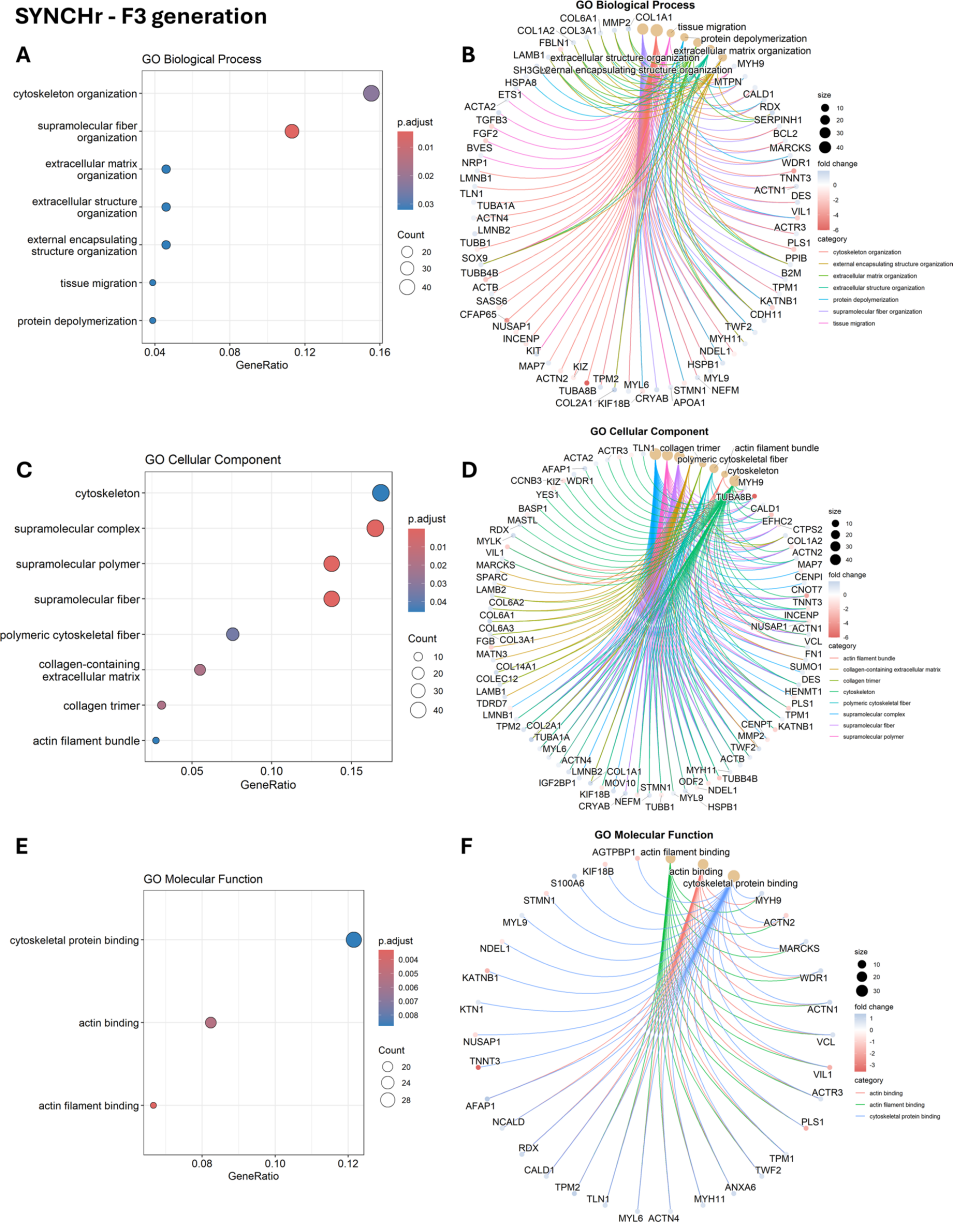
Gene ontology (GO) enrichment analysis of differentially expressed genes (DEGs) in SYNCHr compared to control in F3 generation. (**A**, **C**, **E**): dot plots showing the top 10 enriched biological processes, cellular components and molecular functions, respectively. (**B**, **D**, **F**) Cnet plots showing the relationship between the DEGs and GO terms for biological processes, cellular components and molecular functions, respectively.

Functional information was extracted from the DEG datasets using gene ontology (GO) enrichment analysis. The enriched GO terms were categorized into three groups: biological process (BP), cellular component (CC), and molecular function (MF). The lists of all significant GO terms across all comparisons are provided in Supplementary file S4. Significant enrichment in SYN groups in F2 and F3 was not possible due to the low number of DEGs in these groups. No significant enrichment was observed in the SYNCHs group in the F2 generation, whereas the SYNCHr group showed enriched biological processes primarily related to cellular motility and the regulation of responses to external stimuli. In F3, SYNCHs group resulted in 10 significantly enriched CCs, primarily associated with extracellular matrix structure and chromosomal organization (Fig. 3A-B). Only one MF term, collagen binding, was significantly enriched in the SYNCHs group (Fig. 3C-D). On the other hand, the SYNCHr group showed significant enrichment of BP terms related to cytoskeletal organization, extracellular matrix organization, and tissue migration (Fig. 4A–B). Enriched CC terms in this group were associated with supramolecular structures, including the collagen-containing extracellular matrix, cytoskeletal fibers, and actin filament bundles (Fig. 4C–D). The enriched MF terms included actin binding and cytoskeletal protein binding (Fig. 4E–F).

### KEGG enrichment analysis

**Fig. 5:**
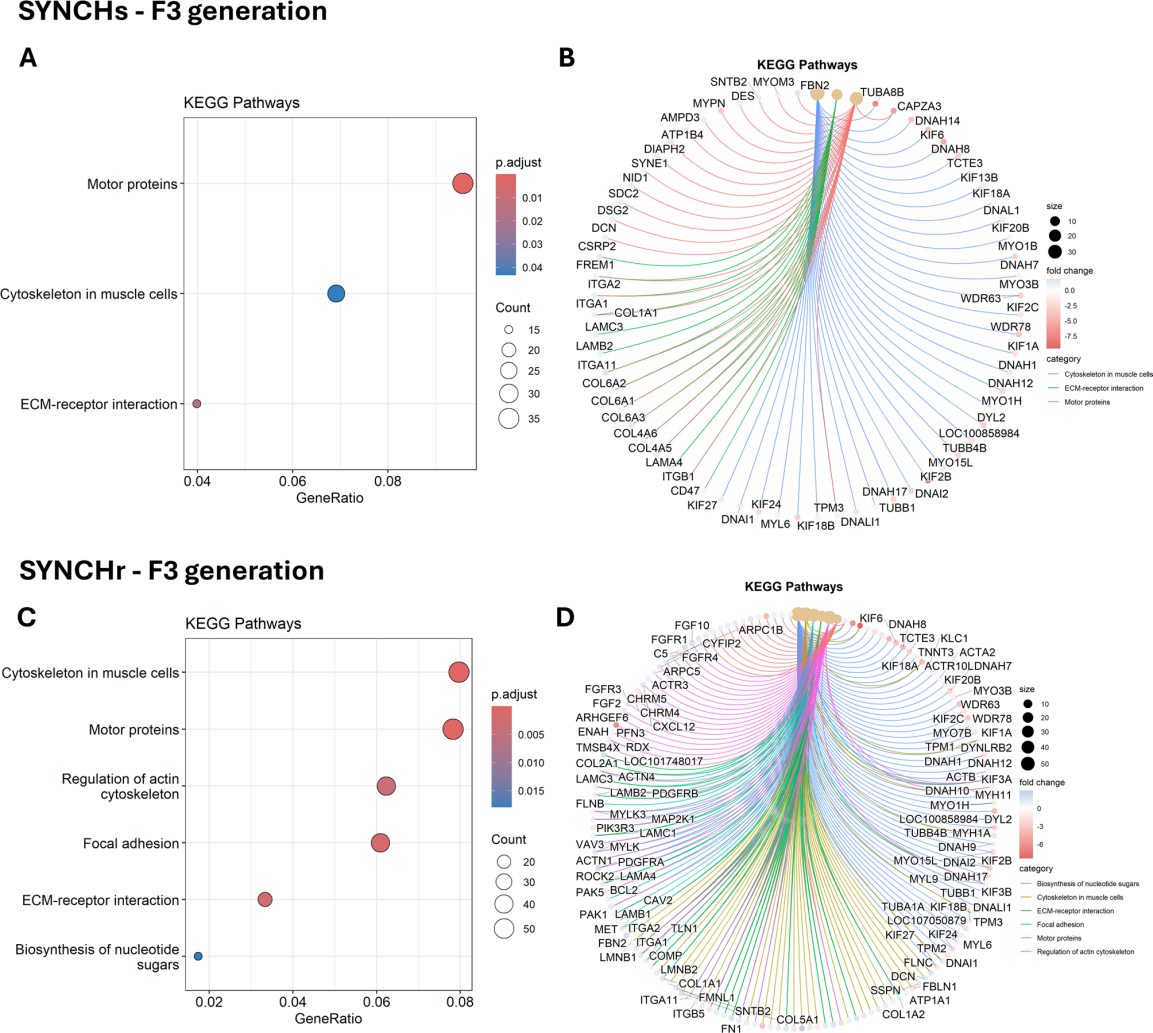
KEGG pathways analysis in F3 generation. (**A**, **C**): significant KEGG pathways in SYNCHs and SYNCHr groups in F3, respectively. (**B**, **D**): Cnet plots showing the relationships between the DEGs and the KEGG pathways in SYNCHs and SYNCHr groups, respectively. Pathway data sourced from the KEGG PATHWAY database – © Kanehisa Laboratories. Used with permission^27–29^.

KEGG pathway enrichment was observed exclusively in the SYNCHs and SYNCHr groups in F3 generation (Fig. 5). Both groups shared enrichment in motor proteins, cytoskeleton in muscle cells, and ECM–receptor interaction pathways. Additionally, the SYNCHr group showed further enrichment in focal adhesion, regulation of actin cytoskeleton, and biosynthesis of nucleotide sugars.

### Validation of sequencing data by RT-qPCR

**Fig. 6:**
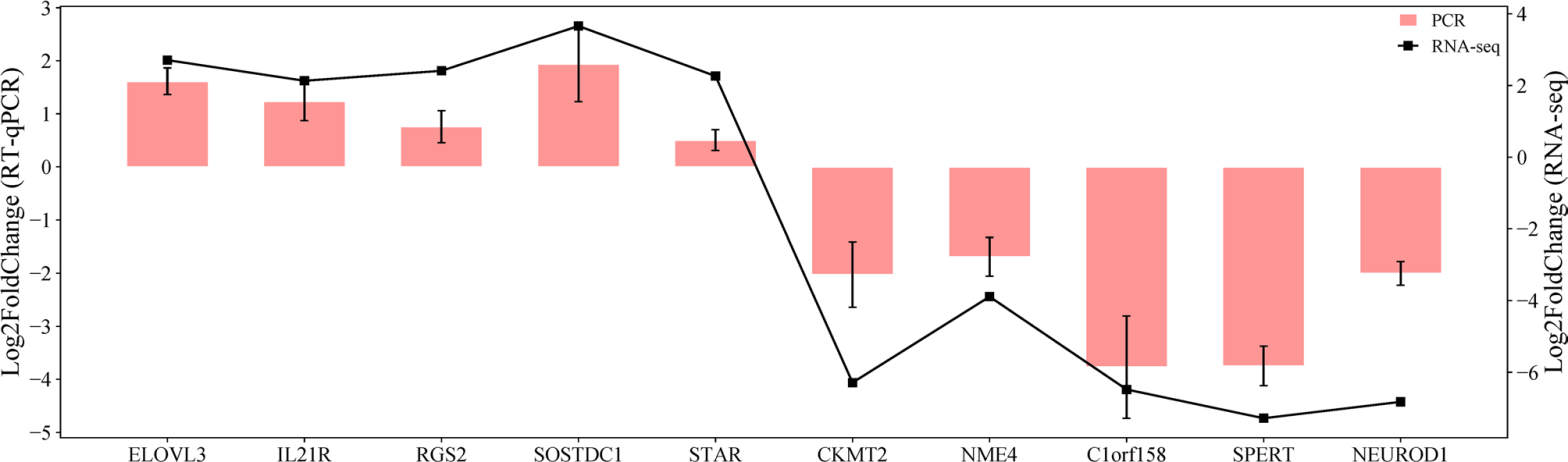
RT-qPCR validation of 10 selected genes. PCR vs. RNA-seq dual y-axis plot for the genes differentially expressed in the SYNCH groups in F3. All data from RT-qPCR analyses were presented as the mean ± standard error of the mean (SEM).

Figure 6 presents the log2 fold change of the ten selected DEGs in the gonadal tissue, analyzed using RT-qPCR and RNA sequencing. RT-qPCR showed the upregulation of *SOSTDC1*, *RGS2*, *ELOVL3*, *STAR* and *IL21R* and the downregulation of *CKMT2*, *C1orf158*, *NME4*, *SPERT* and *NEUROD1* which is consistent with the RNA-sequencing results. Pearson’s correlation test showed a strong, statistically significant positive correlation between RNA-seq and qPCR log2 fold changes (*r* = 0.96, *n* = 10, *p* < 0.001), indicating a high degree of agreement between the two methods. The consistency of the log2 fold change changes (qRT-PCR) and log2 fold changes (RNA-seq) further confirmed the reliability of the RNA-seq data.

### RRBS-based analysis of DNA methylation in synbiotic + choline groups

Since RNA-seq analysis revealed a stronger effect on gene expression following the co-administration of synbiotic and choline compared to synbiotic alone, we performed differential DNA methylation analysis using RRBS data for the SYNCHs and SYNCHr groups compared to their respective controls in the F2 and F3 generations, to assess the effects of in ovo treatment on gonadal methylation profiles. A total of 18 RRBS libraries from male gonads were analyzed, with uniquely mapped reads ranging from about 39.5–66.8% (Supplementary File S5).

**Fig. 7:**
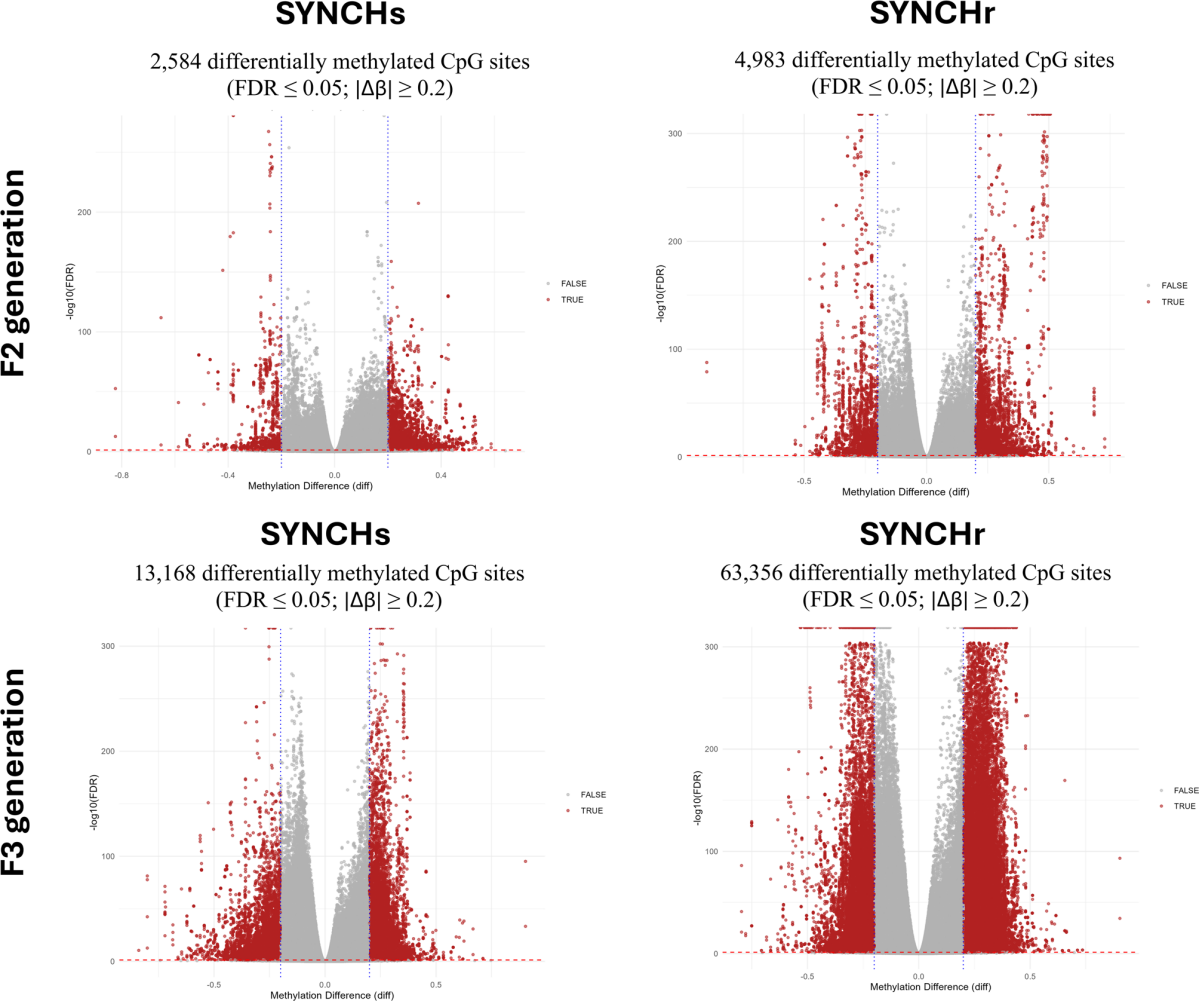
Volcano plots showing differentially methylated CpG loci (DMLs) in SYNCH groups compared to control in F2 and F3 generations (FDR ≤ 0.05, |Δβ| ≥ 0.2). |Δβ| ≥ 0.2: absolute methylation difference of at least 20%.

Differentially methylated loci (DMLs) were identified based on a false discovery rate (FDR) ≤ 0.05 and a methylation difference ≥ 20% (Fig. 7). In the SYNCHs group, 2,584 DMLs were detected in F2, increasing to 13,168 in F3. In the SYNCHr group, 4,983 DMLs were identified in F2, which increased substantially to 63,356 DMLs in F3. These results reflect a pattern similar to the RNA-seq profiles. In the SYNCHs group, 181 differentially methylated regions (DMRs) were detected in F2, of which 171 were annotated to 157 differentially methylated genes (DMGs). By F3, the number of DMRs increased more than fourfold to 786, with 768 annotated, mapping to 629 DMGs. In the SYNCHr group, 258 DMRs were identified in F2, with 247 annotated to 222 DMGs, whereas in F3 this number increased to 2,880 DMRs, of which 2,824 were annotated, corresponding to 1,606 DMGs. In all groups, the majority of DMRs (> 70%) were located in distal intergenic regions (Supplementary file S6). Promoter-associated DMRs (≤ 3 kb from TSS) comprised a higher proportion in SYNCHr groups (14.58% in F2 and 12.78% in F3) compared to SYNCHs groups (5.26% in F2 and 10.16% in F3).

**Fig. 8:**
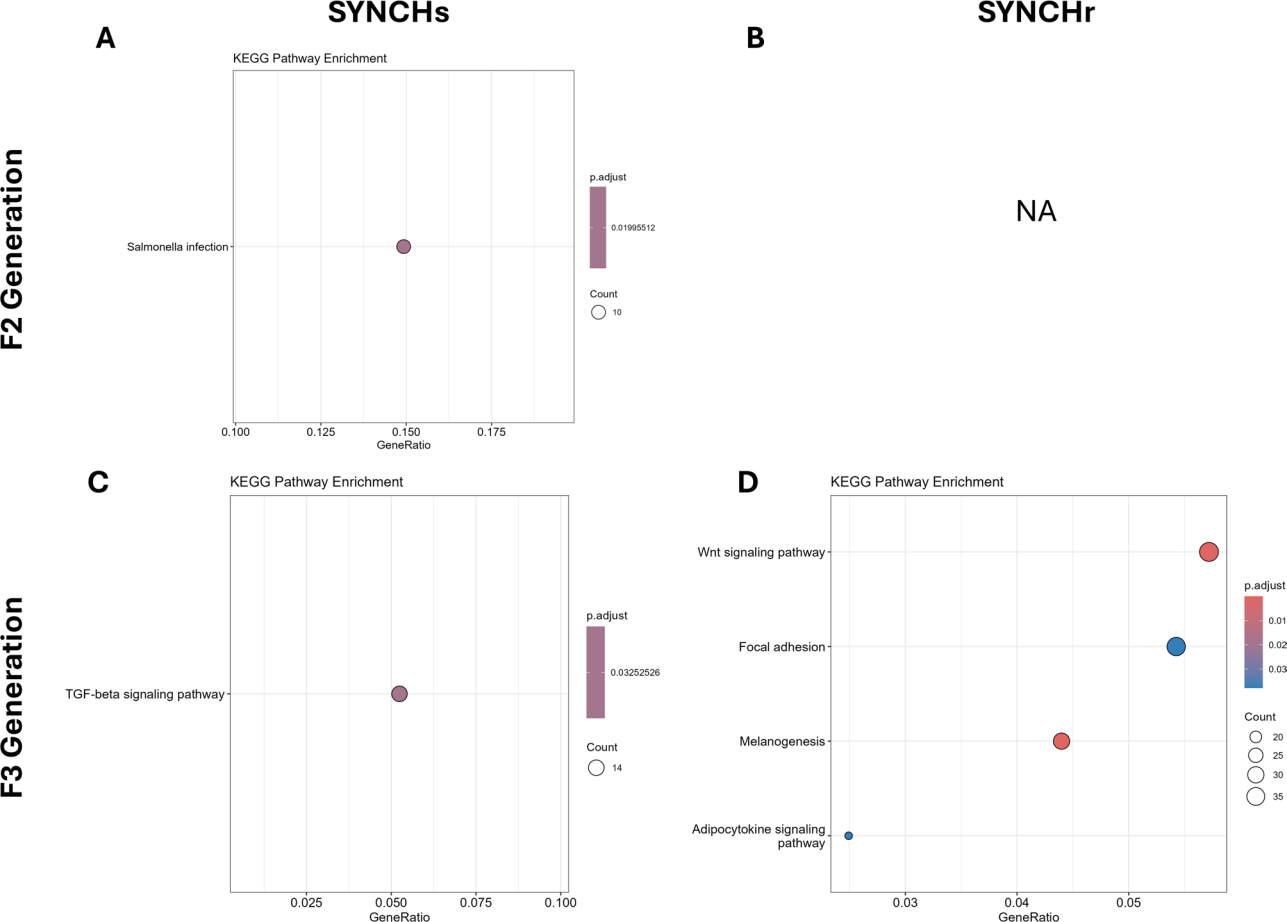
Significantly enriched KEGG pathways associated with differentially methylated genes in the SYNCH groups of F2 and F3 generations. Pathways were identified using an enrichment analysis with a p-value cutoff of 0.05, q-value cutoff of 0.10, and Benjamini-Hochberg (BH) adjustment for multiple testing. Only pathways containing at least three genes were retained. NA: not available (no enrichment). Pathway data sourced from the KEGG PATHWAY database – © Kanehisa Laboratories. Used with permission^27–29^. .

Genes associated with DMRs in the SYNCHs group were enriched in the Salmonella infection KEGG pathway in F2, and the TGF-beta signaling pathway in F3 (Fig. 8). In the SYNCHr group, no pathway enrichment was observed in F2, while in F3, enriched pathways included Wnt signaling, focal adhesion, melanogenesis, and the adipocytokine signaling pathway (Fig. 8). Supplementary file S7 shows the detailed list of enriched pathways with implicated differential methylated genes. No significant enrichment was seen for GO terms in all groups.

**Fig. 9:**
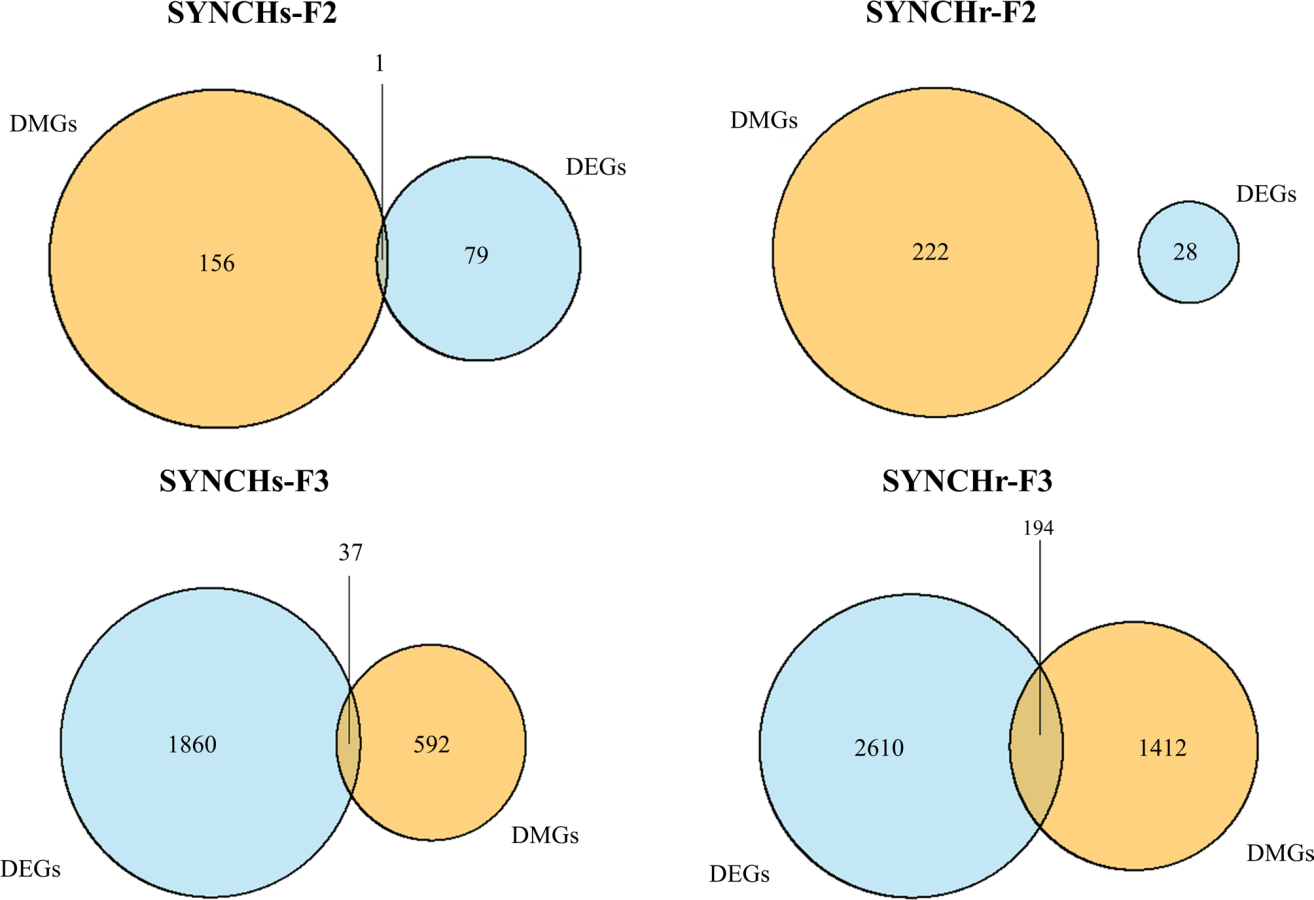
Overlap between differentially methylated genes (DMGs) and differentially expressed genes (DEGs). SYNCHs: group of single injection of synbiotic with choline in F1; SYNCHr: group of repeated injection of synbiotic with choline in F1-F3; F2: second generation; F3: third generation.

Integrative analysis of methylation and transcriptomic data identified concordant methylation-expression changes in 1 gene (1 DMR) in SYNCHs-F2, 37 genes (47 DMRs) in SYNCHs-F3, and 194 genes (306 DMRs) in SYNCHr-F3, while no such overlap was detected in SYNCHr-F2 (Fig. 9). Supplementary File S8 summarizes the overlap between DMGs and DEGs and examines the relationship between the direction of methylation and corresponding gene expression changes in SYNCHs-F2 (Table S1), SYNCHs-F3 (Table S2), SYNCHr-F3 (Table S3). Overall, the integrative results revealed a mixed pattern of methylation–expression associations. In SYNCHs-F2, the only gene with concordant changes was hypermethylated in a distal intergenic region and exhibited upregulated expression. In the SYNCHs group in F3, the majority of DMRs (*n* = 25) showed an inverse correlation with gene expression (e.g., hypermethylation with downregulation or hypomethylation with upregulation), whereas 22 DMRs exhibited concordant changes. In contrast, the SYNCHr group in F3 showed a higher number of DMRs with concordant changes (*n* = 190), while 116 DMRs followed the canonical inverse relationship.

## Discussion

Our analysis of gene expression profiles in male gonads following in ovo stimulation revealed distinct effects, with clear differences between the PS synbiotic alone and PS synbiotic combined with choline. When administered alone to F1 embryos, the synbiotic treatment in the SYNs group induced a modest effect in gene expression in F2 male gonads, which largely diminished by F3 generation, suggesting a weak and fading transgenerational effect. Contrary to our hypothesis, repeated administration of PS synbiotic did not enhance the transcriptional response in F3. However, the single co-administration of PS synbiotic with choline to eggs containing F1 embryos led to a strong effect on gene expression in F3 male gonads. Due to the fact that in each generation all groups were compared to the control, we deduce that the observed effect in generation F3 (in SYNCHs group) can be a response to co-administration of PS synbiotic with choline to eggs containing F1 embryos and therefore can be regarded as a transgenerational effect. Notable, repeated administration of both PS synbiotic with choline supported our hypothesis of a cumulative effect, showing a pronounced transcriptional response in F3 generation, though the effect in F2 remained moderate. To further investigate the effects of synbiotic combined with choline, DNA methylation profiling revealed a pattern consistent with gene expression results, specifically, a substantial increase in the number of DMLs from F2 to F3. Integrative analysis identified a subset of genes in the SYNCHs and SYNCHr F3 groups showing concurrent changes in both methylation and expression, suggesting potential epigenetic regulation in response to the in ovo intervention. Taking this into consideration, it can be deduced that the effects observed in the SYNCHs group of the F3 generation likely represent a transgenerational response initiated by the single in ovo injection of synbiotic and choline in F1 embryos.

The sustained effects seen in the SYNCH groups, in contrast to the SYN groups, may be attributed to different explanations: (1) Choline is an essential nutrient that plays crucial roles in various physiological and epigenetic processes, including DNA methylation, neurotransmitter synthesis, cell membrane integrity, muscle fat metabolism, muscle proteins homeostasis, and the modulation of inflammation and autophagy^39,40^. These mechanisms might lead to more pronounced and lasting effects on gene expression compared to the mechanisms associated with synbiotics. While the components of synbiotics may have wider-reaching effects on the gut microbiota^41^they might not have a potent impact on the gene expression in reproductive tissues such as the gonads to the same degree as choline. (2) The interaction between synbiotics and choline could lead to a synergistic effect, where choline enhances the epigenetic impact of synbiotics, resulting in a more pronounced transgenerational effect as seen in the SYNCHs group. Different types of supplementations can indeed have varying effects, even on the same tissue. For instance, a study by Handy et al. investigated the effects of independent and combined supplementation with nitrate and resveratrol on metabolic adaptations in high-fat-fed male mice^42^. Their main findings highlighted that both supplementations independently improve glucose tolerance and reduce markers of cellular stress. However, when nitrate and resveratrol were co-supplemented, the improvement in glucose tolerance was attenuated.

Several research studies have illustrated the impact of nutriepigenetic substances on male gonadal gene expression and DNA methylation and their transgenerational effects through the male germline. Saito et al. have explored the impact of micronutrient supplementation on gene expression and DNA methylation profiles in the male gonads of Atlantic salmon^17^. Notably, the supplementation influenced the expression of genes associated with three biological pathways in gonads: up-regulation of cytokine receptor interaction and down-regulation of mismatch repair and DNA replication^17^. In terms of DNA methylation, micronutrient supplementation affected the methylation status of genes linked to critical pathways for embryonic development, including cell signaling and synaptic signaling. Chan et al. showed that lifetime exposure of male mice to methyl donor folic acid diets resulted in changes in the DNA methylation, primarily exhibiting hypomethylation, affecting genes involved in neurodevelopmental pathways across F1, F2, and F3 male germ cells^43^. The number of differentially methylated cytosines decreased in F2 sperm compared to F1 but unexpectedly increased in F3 sperms. Although there was no significant retention of inter- and trans-generational inheritance of differentially methylated cytosines, young long interspersed nuclear elements (LINEs) were notably impacted up to the third generation^43^.

In our study, a potential transgenerational effect was observed in the F3 generation following a single injection of PS synbiotic with choline into F1 embryos (SYNCHs group). We found 14 DEGs common between SYNCHs group in F2 and F3 generation. Among these, *PTCHD3* (patched-domain containing 3) is a male germ cell–specific gene, expressed in the midpiece of sperm in mouse, rat, and human, and has been proposed to function as a receptor for Hedgehog (Hh) signaling to regulate sperm development and/or function^44^. However, functional tests revealed that it is not critical for spermatogenesis or fertility in mice^45^. Another shared DEG, *LOC107050879* (also known as kinesin heavy chain *KIF5A*), encodes a motor neuron protein involved in the intracellular transport of organelles, proteins, and RNA, and is predominantly expressed in neurons^46^. However, analysis of mouse testis shows that *KIF5A* is expressed in somatic cells of the testis^47^. *SCARA5* (Scavenger receptor class A member 5) is implicated in iron homeostasis^48^. SCARA5 is expressed in embryonic male gonadal somatic cells, where it mediates ferritin-based iron uptake essential for activating the *Sry* (Sex-determining Region Y) gene^49^. Maternal iron deficiency disrupts this pathway, leading to impaired iron-dependent epigenetic regulation, which in turn causes male-to-female sex reversal by inhibiting Sertoli cell differentiation and proper testis development in mouse embryos^49^. This highlights the essential role of *SCARA5*-mediated iron acquisition in the epigenetic control mechanisms governing male gonadal differentiation^49^. *MIR22* is a microRNA known for its cytoprotective effects, including anti-oxidative, anti-inflammatory, and anti-apoptotic functions^50^. During fetal testicular development in sheep, *MIR22* is upregulated, suggesting a potential role in male gonadal differentiation^51^. This is supported by predictions that *MIR22* represses estrogen signaling pathways, which are commonly associated with ovarian development^51^. In situ hybridization studies have localized *MIR22* expression specifically to Sertoli cells within fetal testicular cords^51^. *CXorf65*, a poorly characterized open reading frame located on the X chromosome (Gene ID: 101748108; *CXorf65* homolog), is highly expressed in the testis of both mice and humans^52^. Its mouse ortholog, *Gm614*, has been shown through knockout studies to impair sperm binding and fertilization, underscoring its functional importance in male fertility^52^. Additionally, *ANKRD60* (Ankyrin repeat domain 60) is classified as reproductive tract-specific in humans and mice, suggesting a potential role in reproduction^53^.

The enrichment was mainly seen in F3 generation for SYNCH groups. The only molecular function affected in the SYNCHs group in the F3 generation is collagen binding, which may be attributed to the effect of synbiotic. Probiotic and synbiotic treatments can accelerate extracellular matrix (ECM) remodeling by stimulating fibroblast activity and enhancing collagen deposition, thereby promoting faster tissue repair and re-epithelialization^54^. Consistent with these findings, CCs associated with collagen and ECM structure were also enriched in the SYNCHs group.

The SYNCHr group of F3 showed enrichment in GO terms mainly related to cytoskeletal and ECM organization. Choline lipids, particularly phosphatidylcholine species with saturated fatty acids, contribute to ECM organization by enhancing membrane rigidity, supporting focal adhesion formation, and facilitating stable cell-ECM interactions^55^. Additionally, probiotic treatments have been shown to support ECM remodeling by stimulating fibroblast activity and increasing collagen deposition^54^.

Both the SYNCHs and SYNCHr groups showed enrichment in similar KEGG pathways, primarily involving motor proteins, cytoskeletal components in muscle cells, and ECM-receptor interactions. Notably, the SYNCHr group also exhibited additional enrichment in pathways related to focal adhesion and nucleotide sugar biosynthesis. The known involvement of choline in membrane biosynthesis, cell adhesion, and one-carbon metabolism lends biological support to the KEGG pathway enrichments observed^56^.

On the other hand, in the SYNCHs group in the F2 generation, DMGs were enriched in the KEGG pathway associated with Salmonella infection, which is indicative of immune system-related processes. Appropriately selected probiotics and prebiotics can exert potent immunomodulatory effects^57^. Additionally, cholinergic signaling can contribute to immune regulation and maintenance of homeostasis^58^. In the F3 generation, DMGs in the SYNCHs group were enriched in the TGF-β signaling pathway. Choline has been shown to induce an anti-fibrotic effect both in vivo and in vitro by regulating the TGF-β1/Smad2/3 and p38MAPK pathways^59^. KEGG enrichment in the SYNCHr group highlighted pathways including Wnt signaling, focal adhesion, melanogenesis, and adipocytokine signaling. Acetylcholine receptors are known to regulate immune-related genes, including those involved in Wnt-mediated host immune response, thereby highlighting a gut-brain-microbial axis driven by cholinergic signaling and Wnt pathway activation^60^. Additionally, dietary choline has been demonstrated to reduce body fat mass gain, prevent adipocyte hypertrophy, and attenuate adipose tissue inflammation, processes regulated by adipocytokines^61^. Acetylcholine and acetylcholinesterase inhibitors, both linked to choline metabolism, have been shown to inhibit light-induced melanogenesis in vitro in melanocytes and ex vivo in mouse skin^62^.

The limitation of this study is the incomplete annotation of the chicken genome, which impacts both gene mapping and downstream analyses^63^. During gene ID conversion (e.g., from gene symbols to ENTREZIDs) for pathway enrichment, a substantial number of genes could not be mapped due to missing or inconsistent entries in public databases. This limitation may have affected the integrative analysis by reducing the apparent overlap between DMR-associated genes and DEGs. Inconsistent gene identifiers likely led to the omission of valid gene matches, thereby underrepresenting potential epigenetic regulation.

## Conclusion

To the best of our knowledge, this study is the first to employ a chicken model to explore the transgenerational effects of in ovo administration of bioactive compounds on reproductive tissues. While transgenerational inheritance remains a debated topic, our findings show that a single exposure of F1 embryos to synbiotic combined with choline (SYNCHs) can induce changes in gene expression and DNA methylation detectable in the F3 generation, supporting the potential occurrence of transgenerational effects of these combined substances. Nonetheless, further additional research, both in vivo and in silico, is required to enhance the identification of intergenerational and transgenerational epigenetic marks responsive to nutritional signals.

## Supplementary Information

Below is the link to the electronic supplementary material.

Supplementary Material 1

Supplementary Material 4

Supplementary Material 5

Supplementary Material 7

Supplementary Material 3

Supplementary Material 8

Supplementary Material 6

Supplementary Material 2

## Data Availability

Sequence data that support the findings of this study have been deposited in the National Center for Biotechnology Information (NCBI) under the primary accession code: PRJNA1142492 for RNA-seq data (http://www.ncbi.nlm.nih.gov/bioproject/1142492) and PRJNA1303698 for RRBS data (https://www.ncbi.nlm.nih.gov/bioproject/PRJNA1303698).
