# Supplementary material for: Transgenerational effects of in ovo stimulation with synbiotic and choline on gonadal tissue across three generations
Source: Sci Rep. 2025 Aug 22;15:30940. doi: 10.1038/s41598-025-16387-6 (PMC12373746; doi:10.1038/s41598-025-16387-6)
Supplement: Supplementary file 7 — Supplementary Material 6 [file 41598_2025_16387_MOESM7_ESM.docx]

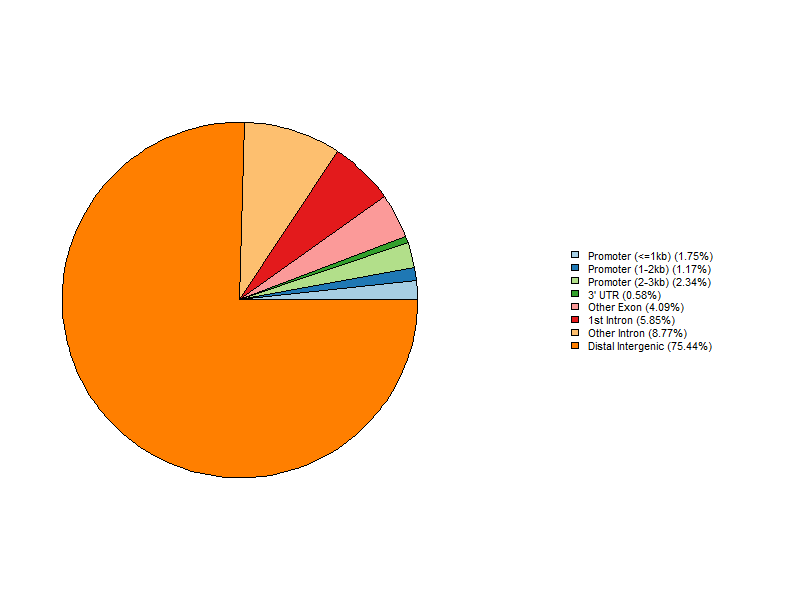


Figure S1. Pie chart showing differentially methylated regions (DMR) genomic distribution in SYNCHs in F2.


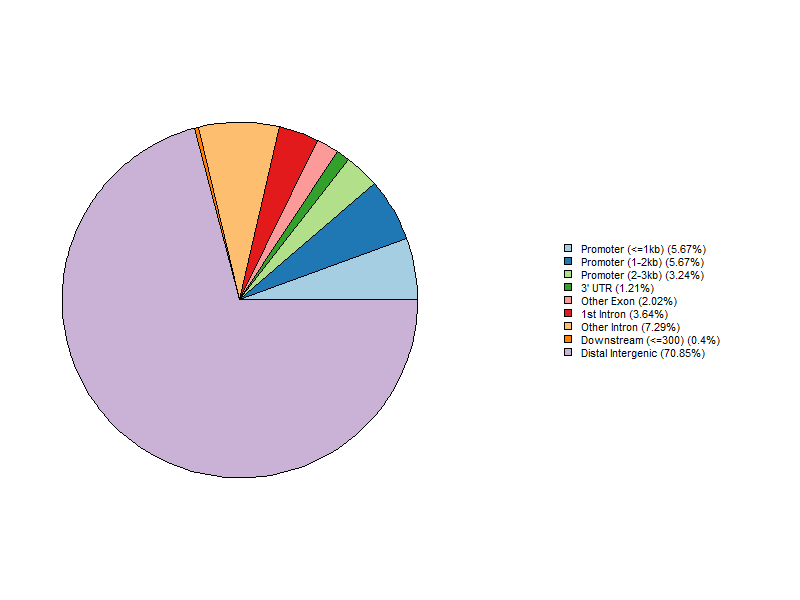


Figure S2. Pie chart showing differentially methylated regions (DMR) genomic distribution in SYNCHr in F2.


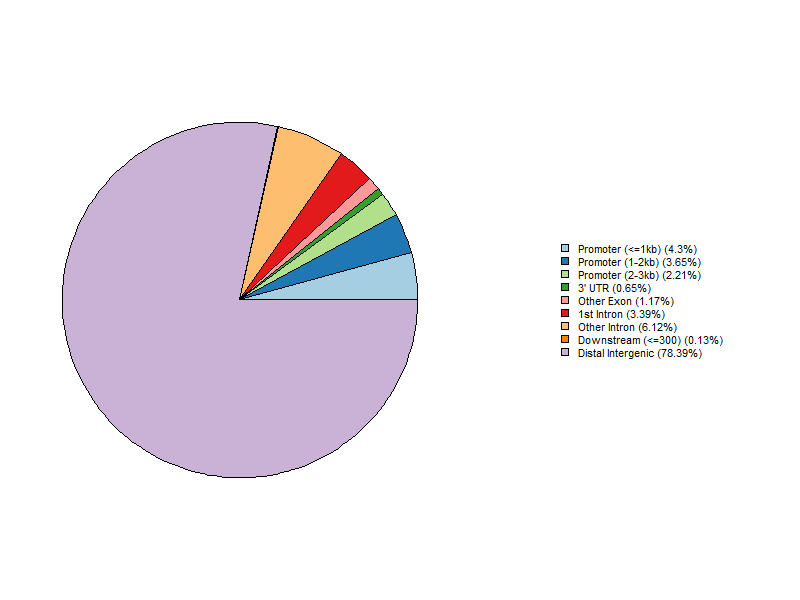


Figure S3. Pie chart showing differentially methylated regions (DMR) genomic distribution in SYNCHs in F3.


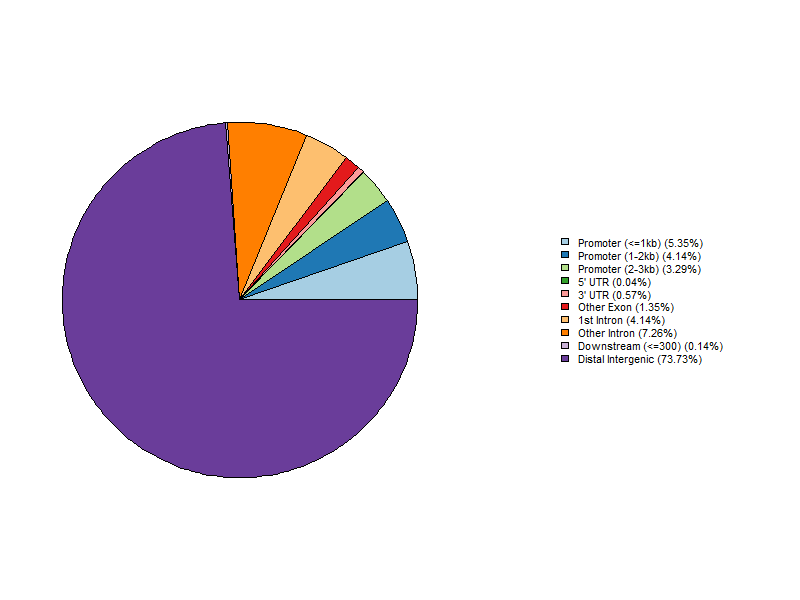


Figure S4. Pie chart showing differentially methylated regions (DMR) genomic distribution in SYNCHr in F3.
